# Poxvirus-Based Active Immunotherapy with PD-1 and LAG-3 Dual Immune Checkpoint Inhibition Overcomes Compensatory Immune Regulation, Yielding Complete Tumor Regression in Mice

**DOI:** 10.1371/journal.pone.0150084

**Published:** 2016-02-24

**Authors:** Susan P. Foy, Barbara Sennino, Tracy dela Cruz, Joseph J. Cote, Evan J. Gordon, Felicia Kemp, Veronica Xavier, Alex Franzusoff, Ryan B. Rountree, Stefanie J. Mandl

**Affiliations:** Bavarian Nordic, Inc., Redwood City, California, United States of America; Ohio State University, UNITED STATES

## Abstract

Poxvirus-based active immunotherapies mediate anti-tumor efficacy by triggering broad and durable Th1 dominated T cell responses against the tumor. While monotherapy significantly delays tumor growth, it often does not lead to complete tumor regression. It was hypothesized that the induced robust infiltration of IFNγ-producing T cells into the tumor could provoke an adaptive immune evasive response by the tumor through the upregulation of PD-L1 expression. In therapeutic CT26-HER-2 tumor models, MVA-BN-HER2 poxvirus immunotherapy resulted in significant tumor growth delay accompanied by a robust, tumor-infiltrating T cell response that was characterized by low to mid-levels of PD-1 expression on T cells. As hypothesized, this response was countered by significantly increased PD-L1 expression on the tumor and, unexpectedly, also on infiltrating T cells. Synergistic benefit of anti-tumor therapy was observed when MVA-BN-HER2 immunotherapy was combined with PD-1 immune checkpoint blockade. Interestingly, PD-1 blockade stimulated a second immune checkpoint molecule, LAG-3, to be expressed on T cells. Combining MVA-BN-HER2 immunotherapy with dual PD-1 plus LAG-3 blockade resulted in comprehensive tumor regression in all mice treated with the triple combination therapy. Subsequent rejection of tumors lacking the HER-2 antigen by treatment-responsive mice without further therapy six months after the original challenge demonstrated long lasting memory and suggested that effective T cell immunity to novel, non-targeted tumor antigens (antigen spread) had occurred. These data support the clinical investigation of this triple therapy regimen, especially in cancer patients harboring PD-L1^neg/low^ tumors unlikely to benefit from immune checkpoint blockade alone.

## Introduction

Poxvirus-based active immunotherapies are in development to treat a variety of cancers. Poxviruses are large DNA viruses that can be engineered to encode tumor-associated antigens such as PSA, HER-2, CEA and MUC-1, as well as immune-stimulatory cassettes, such as the triad of costimulatory molecules (TRICOM) encoding B7.1, ICAM-1 and LFA-3 [1–4]. Nonclinical and clinical studies have demonstrated that these poxvirus-based active immunotherapies generate robust antigen-specific immune responses. These tumor-infiltrating, antigen-specific T cells produce multiple cytokines (particularly high amounts of IFNγ and TNFα), exert cytotoxic activity, and improve the T_eff_:T_reg_ ratio to delay tumor growth [3,5].

Naturally occurring (endogenous) or immunotherapy-induced immune responses are kept in-check by the immune system through engagement of immune checkpoint molecules. Effector T cells simultaneously express multiple inhibitory immune checkpoint molecules such as cytotoxic T-lymphocyte antigen 4 (CTLA-4), programmed death receptor-1 (PD-1), lymphocyte activation gene-3 (LAG-3), and others to control the immune response [6]. While these mechanisms are important to restrict auto-immunity, they can also hinder the development, persistence, and function of desired anti-cancer immunity. Antibodies to block immune checkpoint molecules are being developed and in some indications, approved for clinical use to reverse or prevent the suppression of anti-cancer T cell immune responses [7,8]. Monotherapy with immune checkpoint blockade has yielded remarkable rapid and durable clinical benefit for some cancer patients, ushering a new era of immuno-oncology for cancer treatment.

PD-1 and its binding partners (PD-L1 and PD-L2) represent an important step in immune checkpoint control regulating peripheral T cell responses that enable self-tolerance and prevent auto-immune reactions [9]. In cancer, PD-L1 expression in the tumor microenvironment causes T cell suppression through PD-1 ligation, which leads to tumoral evasion from immune surveillance and therefore resistance. There appear to be two mechanisms for PD-L1 up-regulation in tumors—innate and adaptive resistance. Innate resistance is driven by aberrant oncogenic signaling pathways and results in tumor cells that constitutively express PD-L1 [10,11]. In contrast, adaptive resistance occurs in response to IFNγ produced by tumor-infiltrating T cells provoking PD-L1 upregulation on cells in the tumor microenvironment [12,13]. PD-1 axis blockade confers significant clinical benefit, especially for patients with a pre-existing T cell-inflamed tumor microenvironment characterized by CD8+ and PD-1/PD-L1+ cells [14,15]. Conversely, without an endogenous anti-cancer T cell immune response, as presumed in cancer patients harboring PD-L1^neg/low^ tumors, the immune checkpoint blockade is unfocused and not expected to confer significant clinical benefit [15,16]. We hypothesized that poxvirus-based immunotherapy would drive antigen-specific T cells to the tumor, concomitant with IFNγ production, thus inducing PD-L1 expression in the tumor microenvironment. Therefore, this otherwise productive immune response would be enabled into synergistic anti-tumor efficacy when combined with PD-1 axis blockade.

T cell mediated immune suppression may stem from combined impact of multiple immune checkpoints. Combining PD-1/PD-L1 blockade with LAG-3 inhibition has shown efficacy in preclinical models of infectious disease and cancer [17,18]. The CD4-related transmembrane protein LAG-3 is an immune checkpoint molecule expressed on activated T cells, NK cells, B cells, and plasmacytoid dendritic cells [19–22]. Structurally, LAG-3 is highly homologous to the CD4 T cell co-receptor and binds MHC II [19]. However, its structural interactions with MHC II are different from and are more limited than those of CD4 [23,24]. Early studies showed that LAG-3 affects both CD4 and CD8 T cell function, and plays a role in conventional T cell suppression by Tregs [25–27]. Recently, direct suppression of CD8 T cells by LAG-3 was hypothesized to occur though binding of galectin-3 to glycosylated LAG-3, resulting in cross-linking and activation of the LAG-3 signaling complex [28]. LAG-3 acts independently of and complementary to the PD-1 pathway, and may still inhibit T cell activity when the PD-1 pathway is blocked. We therefore postulated that patients may benefit from combining activation of tumor-specific effector T cells through poxvirus-based active immunotherapy with dual PD-1 and LAG-3 checkpoint inhibition.

In the present preclinical studies, we investigated whether the productive immune response induced by poxvirus-based active immunotherapy resulted in adaptive immune resistance through PD-L1 up-regulation in the tumor microenvironment. Anti-tumor efficacy of MVA-BN-HER2 poxvirus immunotherapy combined with PD-1 blockade, and the effect of these therapies on LAG-3 expression on tumor infiltrating lymphocytes was evaluated. A triple therapy consisting of MVA-BN-HER2 poxvirus therapy, plus anti-PD-1 and anti-LAG-3 dual checkpoint inhibition was explored for optimizing therapeutic efficacy and to assess the durability of responses in mouse models.

## Materials and Methods

### Viruses

MVA-BN-HER2 (Bavarian Nordic, BN, Martinsried, Germany) is a Modified Vaccinia Ankara-based recombinant vector that encodes a modified form of the human epidermal growth factor receptor 2 (HER-2), referred to as HER2 [3]. The modified HER2 comprises the extracellular domains of HER-2 and contains two additional T helper epitopes to enhance immunogenicity [29].

The CV-301 vaccinia vector (CV-301-V) and CV-301 fowlpox vector (CV-301-F) were manufactured under the name PANVAC by the former Therion Biologics Corp (Cambridge, MA, USA). Each poxvirus encodes human carcinoembryonic antigen (CEA) and mucin-1 (MUC-1) with a triad of co-stimulatory molecules (TRICOM) [30]. The infectious unit titers (Inf.U/mL) were determined by flow cytometry [31].

### Tumor Cell Lines

The CT26 murine colon carcinoma cell line expressing human HER-2 (CT26-HER-2) was licensed from the Regents of the University of California [32]. The MC38-MUC-1 cell line was received from the NCI through a cooperative research and development agreement, and was generated from the MC38 colon carcinoma cell line [33,34]. For each cell line, the genetic profile matched the established CT26 and MC38 lines, and cell lines were pathogen free (Idexx Radil, Columbia, MO, USA). Master cell banks and working cell banks were generated and each bank tested positive for HER-2 or MUC-1 by flow cytometry, respectively (not shown).

The CT26-HER-2 and MC38-MUC1 tumor cell lines were treated with recombinant mouse IFNγ at indicated concentrations for 18 hours (0–1000 ng/mL, Millipore, Temecula, CA). Cells were harvested and stained for PD-L1 expression by flow cytometry or immunocytochemistry with the antibodies described below.

### In Vivo Studies

In a solid tumor model, female BALB/c mice (6–8 weeks old, Simonsen Laboratories, Gilroy, CA) were implanted i.d. in the dorsal flank with 1.0×10^5^ CT26-HER-2 cells in 100 μL DPBS. Tumors were measured twice weekly with calipers, and the volume of the tumor calculated according to the following formula: Tumor Volume (mm^3^) = length×width^2^/2. Mice were treated with 1.0×10^7^ Inf.U of MVA-BN-HER2 by tail scarification (t.s. in 7.1 μL TBS) or subcutaneously at the tail base (s.c. in 100 μL) on days 1 and 15. Anti-PD-1 (Rat IgG2a, Clone RMP1-14, Bio X Cell, West Lebanon, NH) or anti-LAG-3 (Rat IgG1, Clone BE0174, Bio X Cell) antibodies were injected i.p. in 100 μL PBS and days 1 and 15 at a dose of 200 μg (~10mg/kg) unless indicated otherwise.

In the experimental lung metastasis model, female BALB/c mice were implanted i.v. with 5.0×10^5^ CT26-HER-2 cells in 300 μL DPBS. Mice were treated with 1.0×10^7^ Inf.U MVA-BN-HER2 (s.c. in 100 μL TBS) on days 4 and 11.

Female C57/BL6 mice were implanted i.v. with 1.0×10^6^ MC38-MUC1 cells in 300 μL DPBS, which forms visible tumors in the lungs after 25 days. Mice were treated with 1E7 Inf.U CV301-V on day 4, and 5E7 Inf.U CV301-F on days 11 and 18 (s.c. in 100 μL 10% Glycerol in PBS).

All animal experiments were performed using protocols approved by the Bavarian Nordic, Inc. Institutional Animal Use Committee. For survival studies, mice were euthanized by CO_2_ asphyxiation when the tumor volume reached 2000 mm^3^ or if they showed signs of distress (abnormal posture, rough coat, abnormal breathing, decreased food or water intake). Mice were monitored daily for signs of distress; no mice were euthanized due to signs of distress and there were no unexpected deaths.

### Flow Cytometry

Solid tumors or lungs/tumors were collected for flow cytometric analysis. Solid subcutaneous tumors or lungs containing experimental pulmonary metastasis were diced to ~1–2 mm^3^ pieces and further digested to single cell suspensions by 1 h incubation at 37°C in DMEM with 10% FBS, 50 U/ml DNAse I and 250 U/mL Collagenase I (Worthington Biochemical Corporation, Lakewood NJ). Red blood cells were lysed with RBC Lysis Buffer (eBioscience).

Antibodies against the following proteins were purchased from BD Biosciences (San Jose, CA): CD45 (Clone 30-F11); BioLegend (San Diego, CA): CD3e (145-2C11), CD4 (RM4-5), CD8a (53–6.7), CD223 (LAG-3, C9B7W), CD274 (PD-L1, 10F.9G2), CD279 (PD-1, 29F.1A12), or eBioscience (San Diego, CA): CD16/CD32 (93). Cells were blocked with anti-CD16/CD32, and stained for surface markers according to standard protocols. All samples were acquired on the BD LSRII or Fortessa and analyzed using FlowJo version 9.6.2 (TreeStar Inc., Ashland, OR).

### Immunohistochemical staining and imaging of tumors

For immunohistochemistry studies, mice were treated as described above, euthanized and perfused with PBS through the left ventricle. Tumors and associated tissue (dermis or lungs) were collected and transferred in fixative (1% paraformaldehyde in phosphate-buffered saline (PBS), pH 7.4). Tissues were rinsed several times with PBS, infiltrated with 30% sucrose, and frozen in OCT compound. Tumor sections, 20-μM thick, were blocked with 5% normal serum in PBS-T (0.3% triton 100X in PBS) and stained according to standard immunohistochemistry protocols [35]. The following primary antibodies were used: (i) PD-L1: rat monoclonal anti-PD-L1 (clone MIH5, diluted 1:500, eBioscience); (ii) CD3 T cells: hamster monoclonal anti-CD3e (145-2C11, 1:200, BD Biosciences) (iii) HER-2 tumor cells: sheep polyclonal anti-HER2 (1:400, Novus Biologicals [Littleton, CO]); (iv) LAG-3: biotin labeled rat anti-LAG-3 (C9B7W, 1:400, BioLegend); (v) CD8 T cells: rabbit monoclonal anti-CD8a (EP1150Y, 1:500, Novus Biologicals); (vi) CD4 T cells: rat anti-CD4 (MCA4635 1:500, AbD Serotec). Secondary antibodies were Alexa Fluor 594 or Alexa Fluor 488-labeled donkey or goat anti-rat, anti-hamster, anti-sheep or anti-rabbit IgG antibody (Jackson ImmunoResearch; all diluted 1:400). For detecting biotin-labeled anti-LAG-3 antibody Alexa Fluor 594-labeled streptavidin was used. Cell nuclei were stained with Vectashield mounting medium containing DAPI (Vector laboratories). Specimens were examined with an Olympus BX60 fluorescence microscope and an Insight Firewire Color Mosaic with IR Filter Camera (Spectra Services, Ontario, NY) at 10 or 20X magnification.

### Area density measurements

An index of area density (proportion of sectional area) was measured in fluorescence microscopic digital images to quantify PD-L1, CD3, CD8, CD4 and LAG-3 in 20-μm-thick sections of tumors with or without treatment. Area density was analyzed in images (10X objective) captured from five regions of tumor in each mouse. The area density was measured at a predetermined threshold with ImageJ [35].

### Statistical Analysis

Statistical analyses were performed as described in the figure legends using GraphPad Prism version 6.01 for Windows (GraphPad Software, La Jolla, CA). Data shown is the mean and standard error of the mean (SEM).

Synergy with the combination therapy in CT26-HER-2 challenged mice was determined by calculating the combination index (CI) using the Chou-Talalay method and CompuSyn Software (www.combosyn.com) [36]. The method requires performing a dose titration of each therapy alone and in combination. The effect at each dose was determined by dividing the average tumor volume of the treatment group by the control group on day 22. A CI < 1 indicates synergism, CI = 1 indicates additive effect, CI > 1 indicates antagonism.

## Results

**Fig 1:**
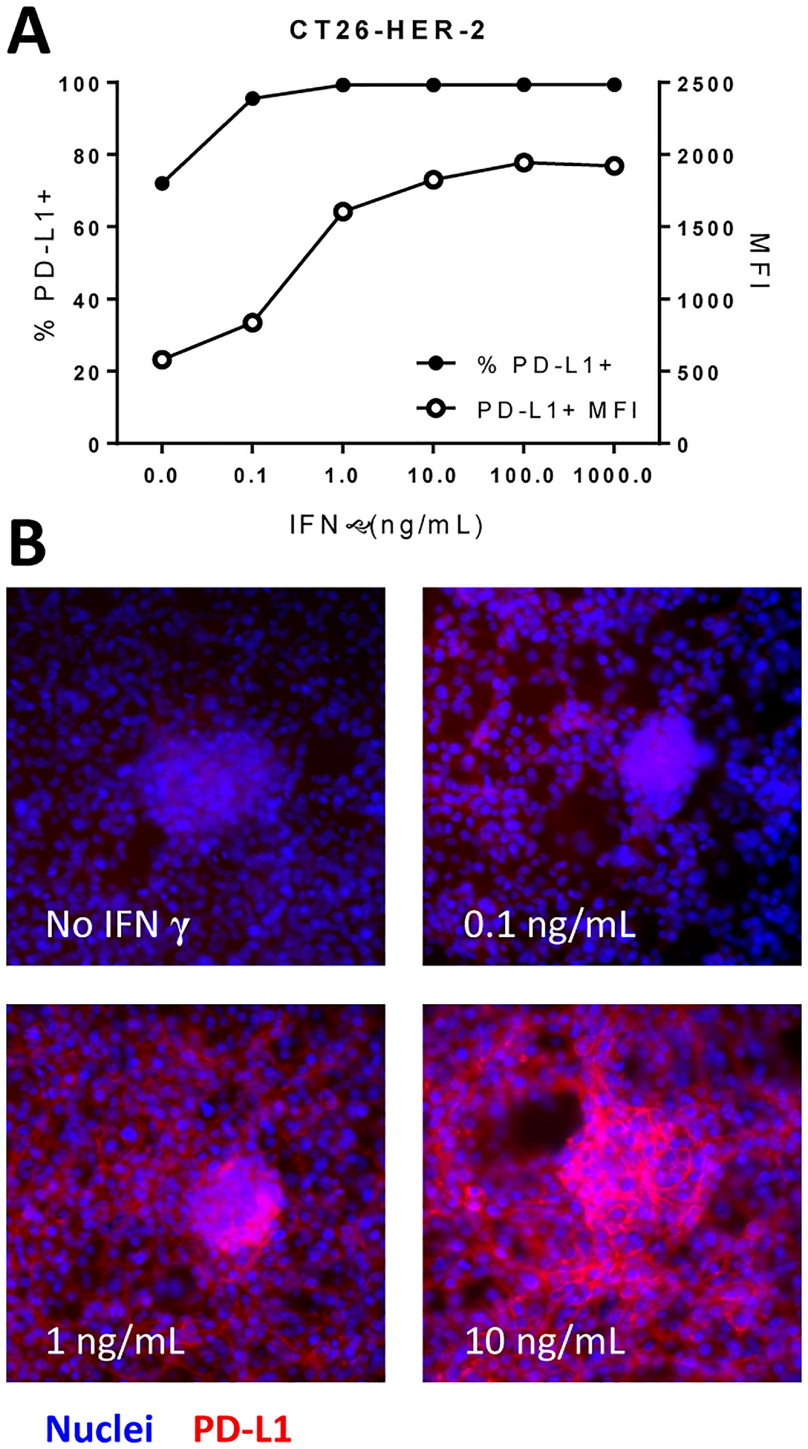
PD-L1 expression following IFNγ stimulation. CT26-HER-2 cells were stimulated with varying concentrations of IFNγ for 18 hours. A) Percent of cells expressing PD-L1 and the mean fluorescence intensity (MFI) by flow cytometry. B) Cells were stimulated with IFNγ for 18 hours at concentrations indicated in each panel then stained for PD-L1 (red) and a nuclei stain (DAPI, blue).

The PD-1/PD-L1 pathway that affords tumor immune evasion is an adaptive resistance mechanism in which PD-L1 expression is induced in the tumor microenvironment by IFNγ producing T cells (TILs). Poxvirus-based active immunotherapies induce infiltration of antigen-specific T cells to the tumor, which produce large amounts of IFNγ after encountering the cognate tumor-antigen [3,5]. We investigated whether poxvirus-based active immunotherapies would increase PD-L1 expression in the tumor microenvironments of transplantable solid tumor or experimental lung metastasis mouse models. Both the CT26-HER-2 and MC38-MUC1 murine tumor cell lines up-regulate PD-L1 in response to IFNγ in a dose-dependent manner (Fig 1 and S1 Fig).

**Fig 2:**
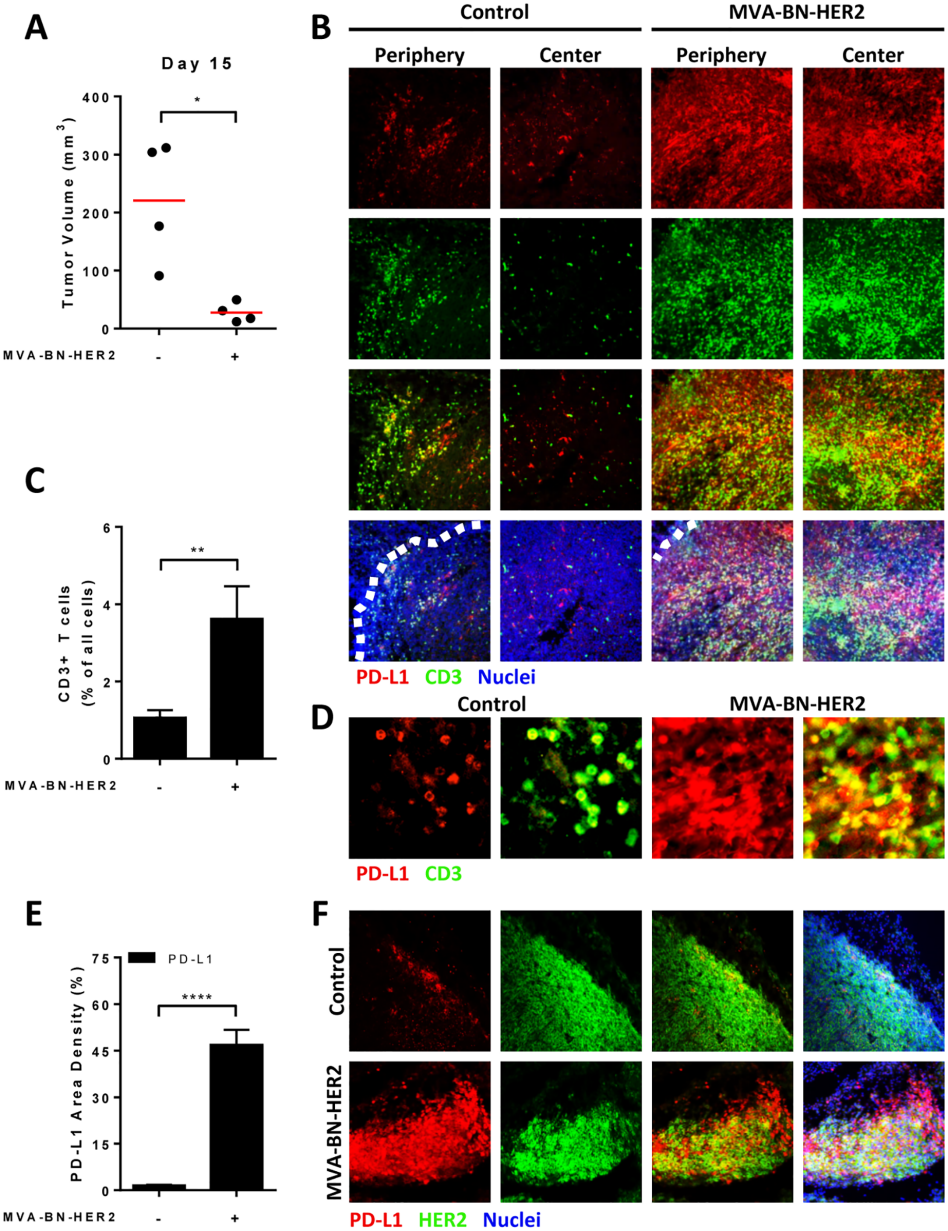
PD-L1 expression in the tumor microenvironment after MVA-BN-HER2 therapy. (**A**) Mice bearing CT26-HER-2 tumors were treated with MVA-BN-HER2 and tumor volume was measured on day 15 compared to control mice (* p<0.05). (**B**) Tumors were collected on day 16 and stained for PD-L1 (red), CD3 (green), and nuclei (DAPI, blue). Representative images show the edge of the tumor (periphery, denoted by white dashed line) and center of the tumor. (**C**) The percent of infiltrating CD3^+^ T was measured by flow cytometry on day 16. (**D**) The area density of PD-L1 on all cells (IHC, **** p<0.0001). (E) Higher magnification images showing the co-localization of PD-L1 (red) and CD3 (green) after treatment. (**F**) Tumor sections were stained for PD-L1 (red), HER2 (green), and nuclei (DAPI, blue). n = 4 mice/group.

As shown in Fig 2A, treatment with the poxvirus-based active immunotherapy, MVA-BN-HER2, significantly delayed tumor growth in a CT26-HER-2 transplantable solid tumor model, and significantly increased CD3+ T cell tumor infiltration compared to control-treated animals (Fig 2B and 2C). Furthermore, PD-L1 expression in the tumor microenvironment was significantly upregulated by treatment with MVA-BN-HER2 immunotherapy (Fig 2D). Immunohistochemical analysis revealed that PD-L1 was upregulated on both HER-2+ tumor cells and on infiltrating CD3+ T cells (Fig 2B, 2E and 2F).

Evidence of increased PD-L1 expression was also observed in experimental lung metastasis models. Challenging mice i.v. with CT26-HER-2 cells results in large tumor burden in lungs and death of untreated mice around day 25 (data not shown). Again, MVA-BN-HER2 immunotherapy significantly delayed tumor growth (S2A Fig) and increased PD-L1 expression on tumor cells (CD45-) compared to control-treated mice (S2B–S2D Fig). Similarly, PD-L1 upregulation also occurred in response to treatment with the CV-301 poxvirus-based active immunotherapy in an MC38-MUC1 experimental lung metastasis tumor model (S3 Fig).

**Fig 3:**
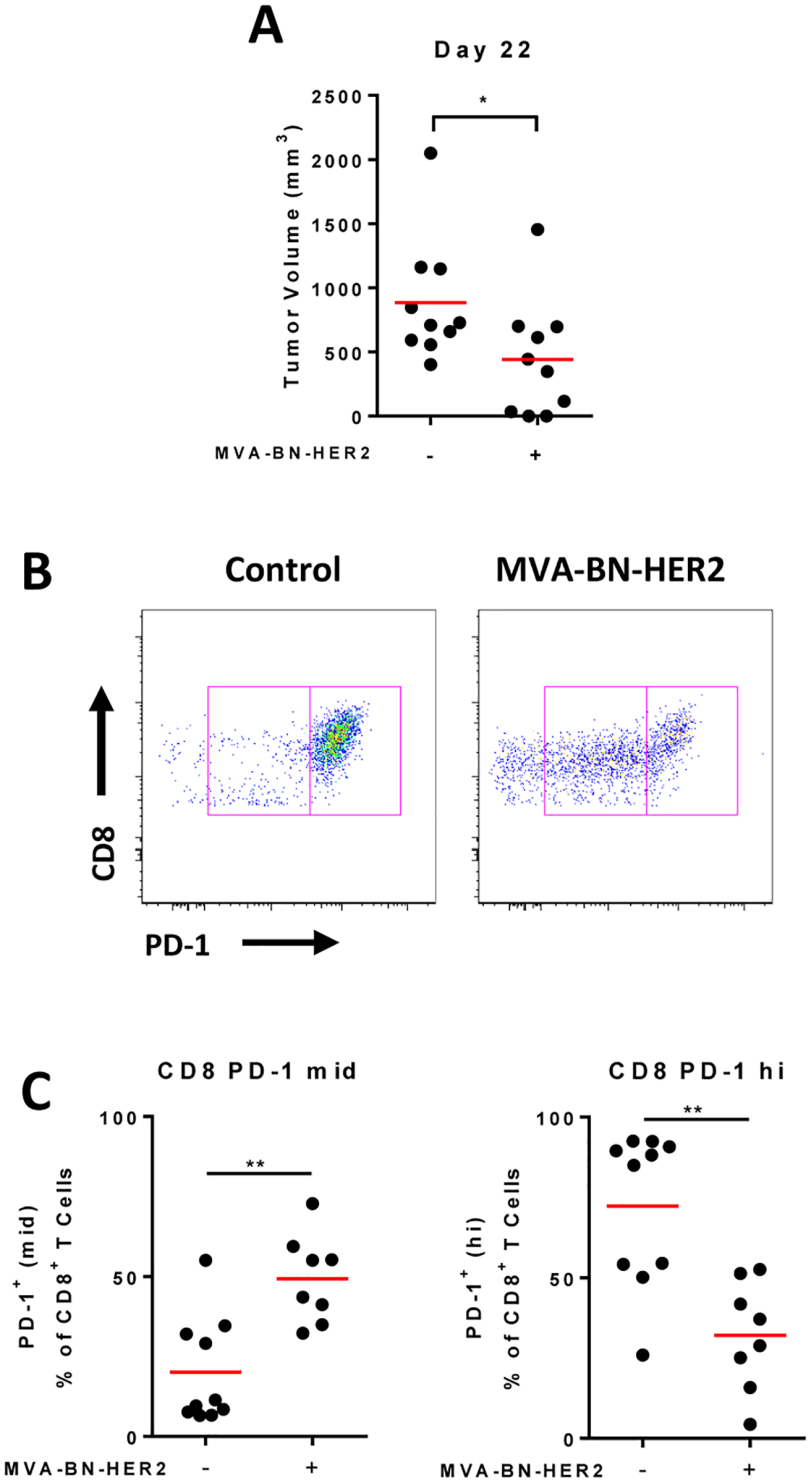
PD-1 expression on CD8 T cells after MVA-BN-HER2 therapy. (**A**) Mice bearing CT26-HER-2 tumors were treated with MVA-BN-HER2 and had a significantly reduced tumor volume on day 22 compared to control mice (* p<0.05). (**B**) Tumors were processed on day 22 and analyzed by flow cytometry. Representative flow cytometry plots show a PD-1 mid (left box) and PD-1 hi (right box) population on CD8+ T cells. (**C**) PD-1 mid (left) and PD-1 hi (right) expression levels on tumor infiltrating lymphocytes. n = 10 mice/group, 2 mice from the MVA-BN-HER2 group were tumor free at day 22.

For PD-L1 mediated tumor immune evasion to promote T cell anergy/exhaustion, tumor-infiltrating CD8 T cells would be expected to express high levels of PD-1. In both solid and lung tumor models (Fig 3A and S2A Fig), the majority of infiltrating CD8 T cells expressed PD-1 (Fig 3B and S3E Fig). However, the level of PD-1 expression on tumor-associated CD8 T cells differed between treated and untreated mice. In control-treated mice, the majority of CD8 T cells expressed high PD-1 (consistent with a more exhausted phenotype), while the CD8 T cell phenotype following MVA-BN-HER2 treatment was dominated by intermediate PD-1 expression (consistent with an activated phenotype, Fig 3C and S2F Fig).

**Fig 4:**
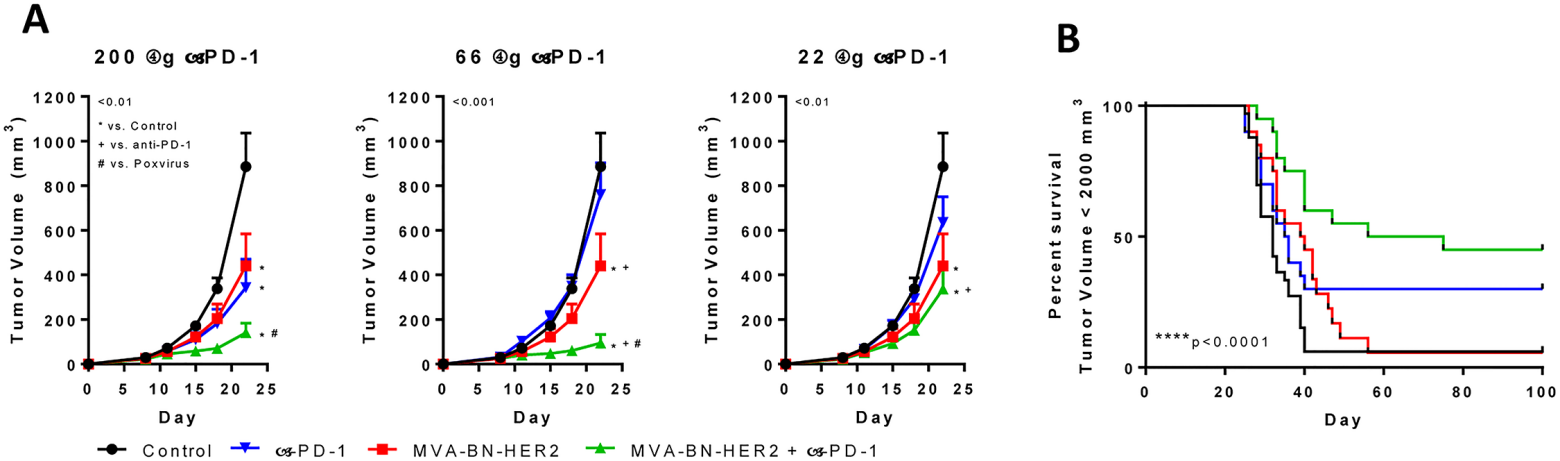
MVA-BN-HER2 synergized with PD-1 to delay tumor growth. (**A**) Tumor volume in mice with MVA-BN-HER2 treatment and anti-PD-1 at varying doses (200 μg, 66 μg, or 22 μg); * p <0.01 vs. control, + p<0.01 vs. anti-PD-1, # p<0.01 vs. MVA-BN-HER2. n = 10 mice/group. (**B**) Survival curves for mice treated in a separate experiment with MVA-BN-HER2 and 200 μg anti-PD-1. Mice were euthanized when the tumor reached 2000 mm^3^; mice alive at day 100 were tumor free, **** p<0.0001. n≈20 mice/group combined from two independent studies.

To counter the potential for limiting productive anti-tumor T cell immunity through PD-1/PD-L1 interactions, we explored the impact of combining poxvirus-based immunotherapy with PD-1 axis blockade to improve anti-tumor efficacy. Mice were challenged by s.c. implantation of CT26-HER-2 tumors, then treated with a fixed dose of MVA-BN-HER2 immunotherapy and/or a range of doses of anti-PD-1 (200 μg, 66 μg, or 22 μg, Fig 4A). Combining MVA-BN-HER2 immunotherapy with 200 μg PD-1 blockade significantly improved the median overall survival and led to complete tumor elimination in 45% of treated animals (Fig 4B). In contrast, MVA-BN-HER2 immunotherapy or PD-1 blockade as monotherapies showed a significant delay in tumor growth with only a moderate increase in the median overall survival (% tumor-free mice improved from 6% (controls) to 10% or 30% after monotherapy with MVA-BN-HER2 immunotherapy or PD-1 blockade, respectively (Fig 4B and S1 Table). Notably, combination therapy still achieved significant anti-tumor efficacy at the lower doses of PD-1 therapy (66 μg and 22 μg), whereas PD-1 blockade alone had little impact on tumor burden (Fig 4A). The improved effect of the combination therapy was strongly synergistic using the Chou-Talalay index for confirmation of therapeutic synergy (combination index (CI) < 0.5, S2 Table).

**Fig 5:**
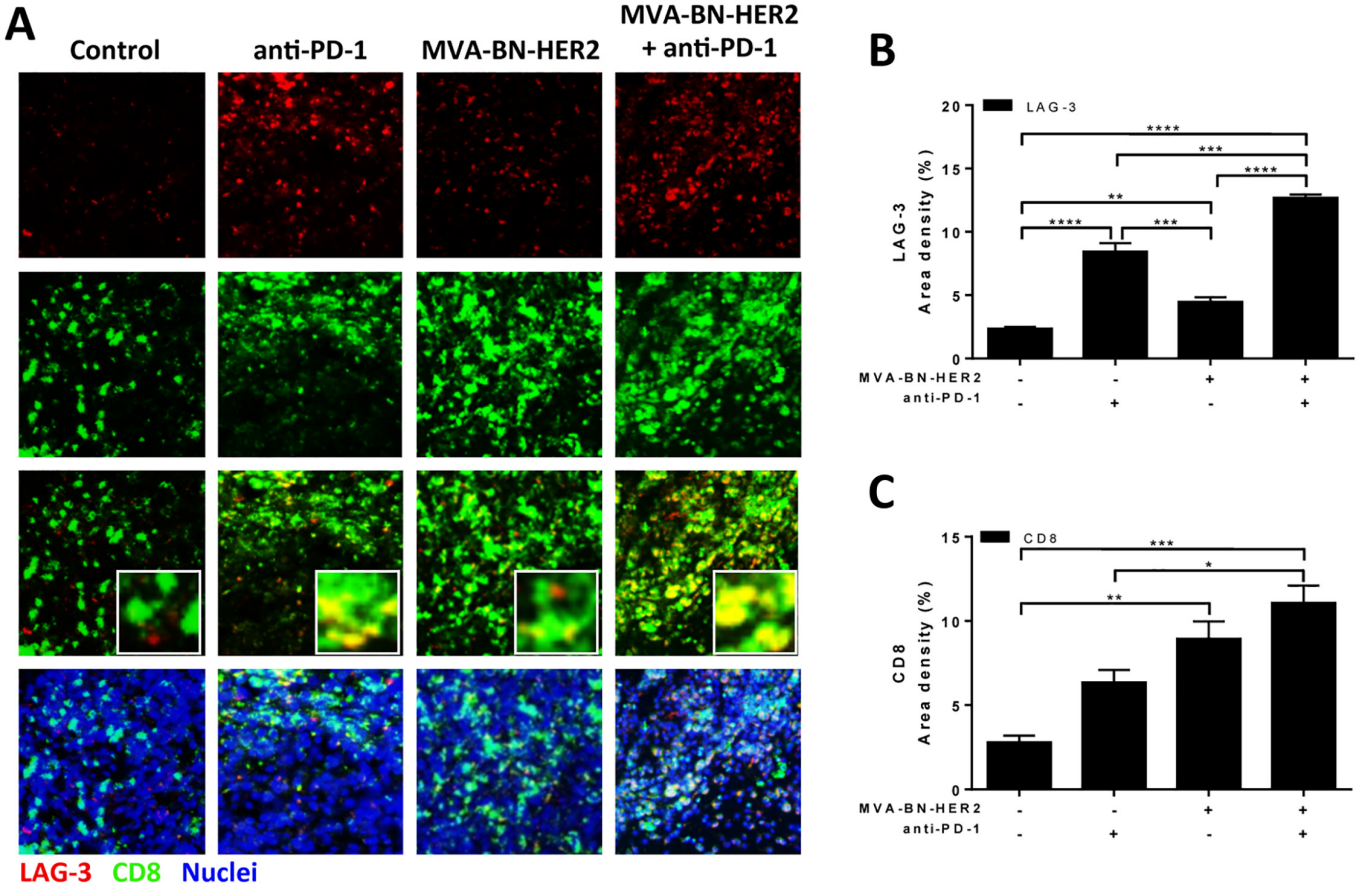
LAG-3 expression in the tumor microenvironment after MVA-BN-HER2 and anti-PD-1 therapy. (**A**) Tumors from mice treated with MVA-BN-HER2 and/or anti-PD-1 were collected on day 16 and stained for LAG-3 (red), CD8 (green), and nuclei (DAPI, blue). (**B**) The area density for LAG-3. (**C**) The area density for CD8+ T cells.* p<0.05, ** p<0.01, *** p<0.001, **** p<0.0001. n = 3–4 mice/group.

Beyond the PD-1/PD-L1 axis of immune suppression pathways, LAG-3 expression has also been shown to impact T cell mediated anti-tumor immune responses. Analysis of intratumoral LAG-3 expression by immunofluorescent microscopy demonstrated that LAG-3 expression co-localized with both CD8+ CD4+ T cells (Figs 5A and 4A). Furthermore, the LAG-3 expression profile and the degree of T cell infiltration in tumors was influenced by the treatment regimen (Fig 5B, 5C and S4B Fig). LAG-3 expression was slightly but significantly increased by MVA-BN-HER2 immunotherapy as compared to control treated tumors. Importantly, the infiltrating CD8 T cells were detected throughout the tumor with MVA-BN-HER2 treatment (Fig 5A and 5C). In contrast, anti-PD-1 monotherapy triggered significantly higher levels of LAG-3 expression (Fig 5B) than MVA-BN-HER2 therapy but T cells remained confined to the tumor periphery. Combining MVA-BN-HER2 immunotherapy with PD-1 blockade resulted in high LAG-3 expression on CD8 T cells and CD8 T cell infiltration throughout the tumor (Fig 5A) and co-localization of CD4 and LAG-3 in patches of the tumor (S4A Fig). Increased tumor infiltration by CD4 T cells was observed in both groups treated with MVA-BN-HER2 but was only significant in the combination treatment group (S3B Fig).

**Fig 6:**
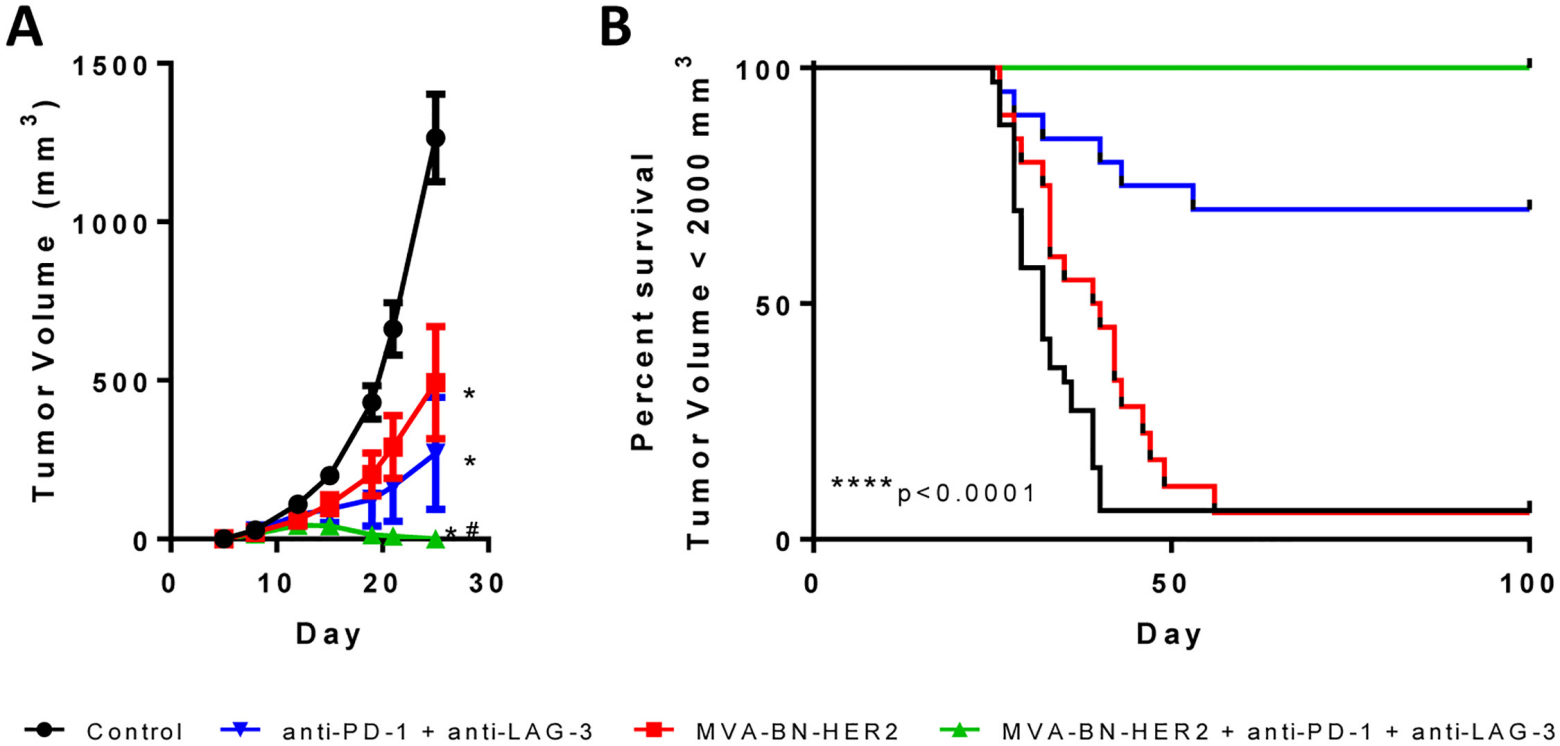
Combining MVA-BN-HER2 with anti-PD-1 and anti-LAG-3 dual checkpoint inhibition led to complete tumor regression. (**A**) MVA-BN-HER2 and/or anti-PD-1 and anti-LAG-3 dual checkpoint inhibition significantly reduced the average CT26-HER-2 tumor volume by day 25, * p<0.0001 vs. control, # p<0.001 vs. MVA-BN-HER2. (**B**) Survival curves for mice with a tumor volume <2000 mm^3^; mice alive at day 100 were tumor free, **** p<0.0001. n≈20 mice/group combined from two independent studies.

**Fig 7:**
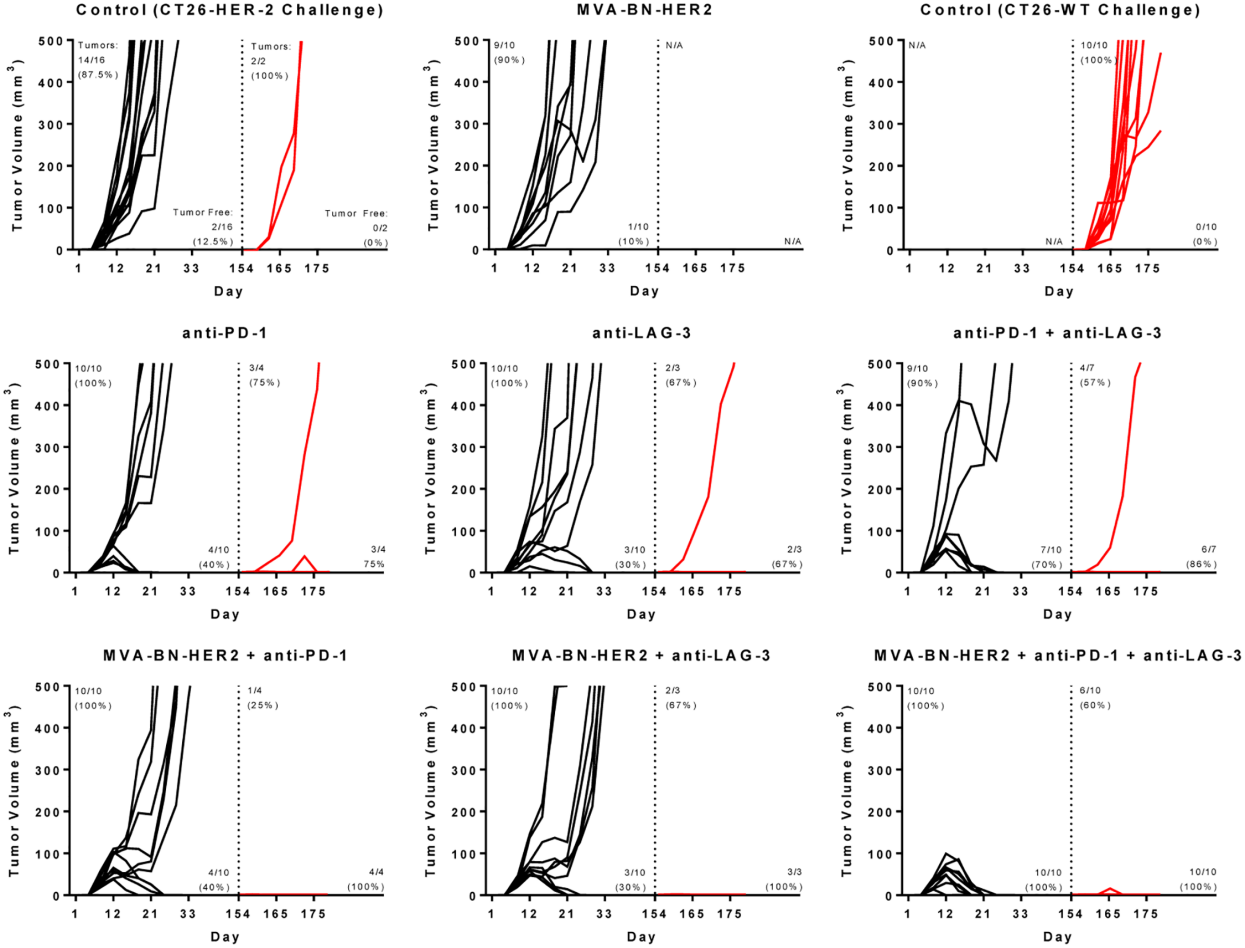
MVA-BN-HER2 therapy combined with PD-1 and/or LAG-3 resulted in durable response with antigen spread. Mice were implanted with CT26-HER-2 tumor cells (black lines) and treated with MVA-BN-HER2, anti-PD-1, and or anti-LAG-3 as indicated in the figure. Tumor free mice were re-challenged with CT26-WT cells not expressing HER-2 (red lines), 154 days after the original challenge (dashed line). Ten additional naïve mice were challenged only with CT26-WT cells as a positive control. The numbers of mice with measurable or palpable tumors is indicated in the top left of each graph, while the numbers of tumor free mice is indicated in the bottom right of each graph. Arrows indicate days of treatment.

These data led to the hypothesis that combination of poxvirus-based immunotherapy with dual PD plus LAG-3 checkpoint inhibition would result in a highly efficacious combination treatment regimen that would trigger a robust and unconstrained tumor-infiltrating antigen-specific T cell response. Consistent with this hypothesis, comprehensive and durable tumor regression was observed in 100% of mice treated with this triple combination therapy (Fig 6A and 6B). Strikingly, tumors were observed to grow in size following initiation of the combination therapy, yet regressed completely upon the second administration two weeks after the first dose (Fig 7, bottom right panel). The complete efficacy of triple combination therapy was greater than anti-PD-1 and anti-LAG-3 immune checkpoint blockade or MVA-BN-HER2 immunotherapy plus anti-PD-1 (Fig 4) or MVA-BN-HER2 immunotherapy plus LAG-3 blockade (S5 Fig). Mice that rejected their tumor after any treatment remained tumor-free more than 5 months after the initial tumor challenge (Fig 7, black lines).

To test whether the targeting and killing of tumors by immune responses were durable and expanded to antigen spread T cell responses, mice that rejected the CT26-HER-2 tumors were subsequently re-challenged with the parental CT26-WT tumor cell line that does not express HER-2 (Fig 7, red lines). As expected, naïve mice succumbed to challenge with CT26-WT tumors. Of mice previously treated with MVA-BN-HER2 and immune checkpoint inhibition, although 53% (9/17) showed evidence of a palpable tumor upon re-challenge within the first 10–15 days post challenge, 100% (17/17) of the mice ultimately rejected the tumors within three weeks. In mice treated with any combination of immune checkpoint inhibitors (anti-PD-1, anti-LAG-3, or anti-PD-1 and anti-LAG-3) but not MVA-BN-HER2, 64% (9/14) grew palpable tumors and 79% (11/14) rejected the re-challenge.

## Discussion

Poxvirus-based active immunotherapy results in significant antitumor immunity characterized by robust CD8 T cell infiltration of the tumor [3,5,37,38]. The studies in this report show that this is also accompanied by significant upregulation of PD-L1 expression in the tumor microenvironment, a known adaptive resistance mechanism that occurs in response to IFNγ produced by tumor-infiltrating T cells [12,13]. When MVA-BN-HER2 immunotherapy was combined with PD-1 blockade, synergistic anti-tumor efficacy was observed, and in 45% of mice the tumors regressed completely. Despite the observed synergy from combination therapy, tumor growth still occurred in the other half of the treated mice. Immunohistochemistry revealed a PD-1 blockade-driven increase in LAG-3 expression on T cells as a potential mechanism of this incomplete response. Notably, when MVA-BN-HER2 immunotherapy was combined with dual PD-1 and LAG-3 immune checkpoint blockade in subsequent experiments, complete tumor regression was observed in all mice. Furthermore, all mice successfully rejected a challenge with tumors that did not express the original tumor antigen 6 month after the original challenge demonstrating that antigen spread had occurred and the observed anti-tumor immune response was durable.

Because PD-L1 upregulation is a mechanism of tumoral adaptive resistance, the observed PD-L1 upregulation in the tumor microenvironment following poxvirus-based active immunotherapy treatment is interpreted as the evasion response to activated cytotoxic CD8 T cells producing IFNγ in high amounts [5,37,39]. These data corroborate evidence from preclinical studies demonstrating that tumors do not upregulate PD-L1 expression in mice lacking T cells or in IFNγ-knockout mice [13]. Elevated PD-L1 expression as a tumor immune evasion mechanism to suppress the activity of tumor-infiltrating, IFNγ-producing T cells was also demonstrated in humans [12]. Recent clinical studies demonstrated a correlation between PD-L1 expression with the presence of TILs [40]. Indeed, PD-1/PD-L1 inhibition appears to provide higher clinical benefit for those patients with PD-L1 positive tumors [41,42]. Together, these correlations suggest that patients with an endogenous or pre-existing tumor-specific T cell immune response may be most likely to benefit from PD-1/PD-L1 axis blockade. However, this leaves a high unmet need for patients with PD-L1^neg/low^ tumors. The preclinical data presented here suggest that these patients may benefit from PD-1 axis blockade if combined with poxvirus-based active immunotherapies that provoke a productive tumor-infiltrating CD8 T cell response. These data further suggest that the evolution of tumors from PD-L1^neg/low^ to PD-L1^hi^ may be useful as a biomarker for the emergence of productive anti-tumor T cell immunity.

In addition to providing a pro-inflammatory immune response together with PD-L1 upregulation in the tumor microenvironment, poxvirus-based active immunotherapy resulted in increased numbers of CD8 T cells expressing intermediate levels of PD-1 (PD-1^mid^). PD-1^mid^ T cells are generally considered more potent at lysing target cells and producing higher amounts of IFNγ and TNFα than PD-1^hi^ cells [43]. In contrast, there were significantly more CD8 T cells that were PD-1^hi^ in the control group. PD-1^hi^ T cells are generally defective in their ability to produce cytokines against target cells and are unable to be rescued by PD-1 immune checkpoint blockade [43,44]. Importantly, the induction of this functional immune response characterized by activated T cells expressing PD-1^mid^ and PD-L1 expression in the tumor microenvironment provided the foundation for synergistic therapy by combination with PD-1 axis blockade. Importantly, it also allowed for the reduction in the dose of anti-PD-1 monoclonal antibody used. Indeed, strong therapeutic synergy was still seen at doses where PD-1 blockade alone showed no effect on tumor growth. This suggests, that at low doses PD-1 blockade is acting to further enhance a functional immune response driven by the poxvirus-based immunotherapy. In contrast, PD-1 blockade alone had little therapeutic benefit with decreasing doses. This could be due to the lack of an endogenous immune response as demonstrated by overall lower numbers of CD8 T cell in the tumor.

While poxvirus-based immunotherapy resulted in PD-1^mid^ CD8 T cells infiltrating into the tumor, we observed a moderate increase of LAG-3 expression in tumor infiltrating CD8 T cells following poxvirus-based immunotherapy alone, and an even greater increase following PD-1 blockade. This was especially apparent when the therapies were combined due to increased infiltration of CD8 T cells into the tumor. These findings agree with previous work showing that vaccination with vaccinia virus elevates intracellular LAG-3 expression in CD8 T cells [27]. Furthermore, they highlight the need to address multiple compensatory immune responses for immunotherapy with immune checkpoint inhibitors, since full therapeutic benefit occurred only when poxvirus-based immunotherapy was combined with dual PD-1 and LAG-3 blockade. CD8 T cells that express LAG-3 can still produce effector cytokines, and cells that co-express mid-levels of PD-1 and high levels of LAG-3 are more functional and produce more IFNγ, TNFα, and CD107 than cells that are PD-1^hi^ or cells that co-express PD-1^low^ and LAG-3 [43]. However, though functional, the proliferative capacity of LAG-3^+^ T cells may be limited, as LAG-3 negatively regulates cell cycle progression of CD8 T cells [27,45]. Thus, one role of LAG-3 blockade may be to increase proliferation of the antigen-specific CD8 TILs, while PD-1 blockade prevents T cell death or anergy through tumor cell PD-L1 ligation.

Poxvirus-based active immunotherapy initially targets specific tumor antigens encoded by the viral vector (e.g. PSA, HER-2, CEA or MUC-1); however, T cell-mediated tumor killing holds the potential to reveal antigen spread T cell responses to *de novo* patient-specific antigens (also known as private antigens or neoantigens). The successful rejection of CT26-WT tumors six months after the initial CT26-HER-2 challenge by mice treated with MVA-BN-HER2 and any immune checkpoint inhibitor demonstrates that the initial productive immune response was durable and had expanded to additional non-targeted endogenous tumor antigens. Antigen spread is thought to play a critical role in successful immunotherapy as the immune system adapts to target novel tumor antigens as well as restricts tumor evasion to therapy. These data corroborate previous findings of antigen spread T cell responses in pre-clinical and clinical studies with poxvirus-based active immunotherapies targeting HER-2, CEA, or PSA [3,5,38,39]. Importantly, these findings further highlight the plasticity and long term durability of productive T cell immunity once tumor-specific killing has been activated.

The preclinical data presented here demonstrate the curative potential of poxvirus–based active immunotherapy in combination with dual checkpoint inhibition using anti-PD1 and anti-LAG-3 antibodies and warrant clinical investigation. In fact, poxvirus-based immunotherapy could be the foundation for improving efficacy of cancer immunotherapy therapy employing immune checkpoint inhibitors in general. Men with metastatic castration resistant prostate cancer (mCRPC) were treated with the poxvirus-based active immunotherapy PROSTVAC and escalating doses of the immune checkpoint inhibitor Ipilimumab (anti-CTLA-4) in a Phase 1 trial [46]. The mOS (31.6 months) from the combined cohorts of PROSTVAC plus any dose of Ipilimumab was notably longer than the mOS of mCRPC patients from the randomized Phase 2 study of PROSTVAC (25.1 months) [47,48]. Furthermore, approximately 20% of patients at the highest dose tested (10 mg/kg) remain alive at 80 months [49]. These clinical data together with the preclinical studies in this report support further clinical investigation of poxvirus-based active immunotherapy with immune checkpoint blockade to address the high unmet need for cancer patients who do not respond to immune checkpoint blockade alone.

## Supporting Information

S1 Fig PD-L1 expression following IFNγ stimulation. MC38-MUC1 cells were stimulated with varying concentrations of IFNγ for 18 hours. A) Percent of cells expressing PD-L1 and the mean fluorescence intensity (MFI) by flow cytometry. B) Cells were stimulated with IFNγ for 18 hours at concentrations indicated in each panel then stained for PD-L1 (red) and a nuclei stain (DAPI, blue).
(TIF)

S2 Fig PD-L1 expression in tumors following MVA-BN-HER2 poxvirus-based immunotherapy in an experimental lung metastasis model. BALB/c mice were implanted with CT26-HER-2 cells (i.v.) on day 1 and treated with MVA-BN-HER2 (1E7 Inf.U) on days 4 and 11. A) Lung mass and tumor burden on day 15. B) Representative Mean Fluorescence Intensity (MFI) in control and MVA-BN-HER2 treated mice, C) Average MFI (n = 5 mice/group). ** p<0.01. D) 20 μm lung and associated tumor section with staining for HER-2 (green), PD-L1 (red) and Nuclei (blue, DAPI). E) Representative flow cytometry from control or MVA-BN-HER2 treated mice on day 15 with a CD8+ PD-1^mid^ population (left box) and a CD8+ PD-1^hi^ population (right box), F) Average CD8+ PD-1^mid^ and PD-1^hi^ expression in the lungs/tumor on day 15.
(TIF)

S3 Fig PD-L1 expression in tumors following PANVAC poxvirus-based immunotherapy in an experimental lung metastasis model. C57/BL6 mice were implanted with MC38-MUC1 cells (i.v.) on day 1 and treated with PANVAC-V (1E7 Inf.U) on day 4 and PANVAC-F (5E7 Inf.U) on days 11 and 18. On day 25 lungs/tumors were collected and stained for H&E or PD-L1.
(TIF)

S4 Fig LAG-3 and CD4 expression in the tumor microenvironment after MVA-BN-HER2 and anti-PD-1 therapy. (**A**) Tumors from mice treated with MVA-BN-HER2 and/or anti-PD-1 were collected on day 16 and stained for LAG-3 (red), CD4 (green), and nuclei (DAPI, blue). (**B**) The area density for CD8+ T cells.* p<0.05. n = 3–4 mice/group.
(TIF)

S5 Fig MVA-BN-HER2 and anti-LAG-3 combination therapy. BALB/c mice were implanted with CT26-HER-2 cells on day 1 (i.d.) and treated with MVA-BN-HER2 (1E7 Inf.U) and/or anti-LAG-3 (200 μg) on days 1 and 15. A) Tumor growth with MVA-BN-HER2 and anti-LAG-3 combination therapy. C) Survival from two independent studies. Statistical significance was determined by: a two-way RM-ANOVA with Tukey’s multiple comparison test for tumor growth or a Log-rank test for survival. * p<0.0001 vs. control.
(TIF)

S1 Table Median Overall Survival (mOS) and % Tumor free mice. NR = not reached. BALB/c mice were implanted with CT26-HER-2 cells on day 1 (i.d.) and treated with MVA-BN-HER2 (1E7 Inf.U), anti-PD-1 (200 μg), or anti-LAG-3 (200 μg) on days 1 and 15. Survival based on tumor volume of 2000 mm^3^.
(DOCX)

S2 Table MVA-BN-HER2 synergized with PD-1 to delay tumor growth. BALB/c mice were implanted with CT26-HER-2 cells on day 1 (i.d.) and treated with MVA-BN-HER2 and anti-PD-1 at doses indicated in the tables. Tumor growth inhibition was calculated from the untreated control. A combination index was calculated with the using the Chou-Talalay method and CompuSyn Software.
(DOCX)
