# Supplementary figures and images for: Poxvirus-Based Active Immunotherapy with PD-1 and LAG-3 Dual Immune Checkpoint Inhibition Overcomes Compensatory Immune Regulation, Yielding Complete Tumor Regression in Mice
Source: PLoS One. 2016 Feb 24;11(2):e0150084. doi: 10.1371/journal.pone.0150084 (PMC4765931; doi:10.1371/journal.pone.0150084)

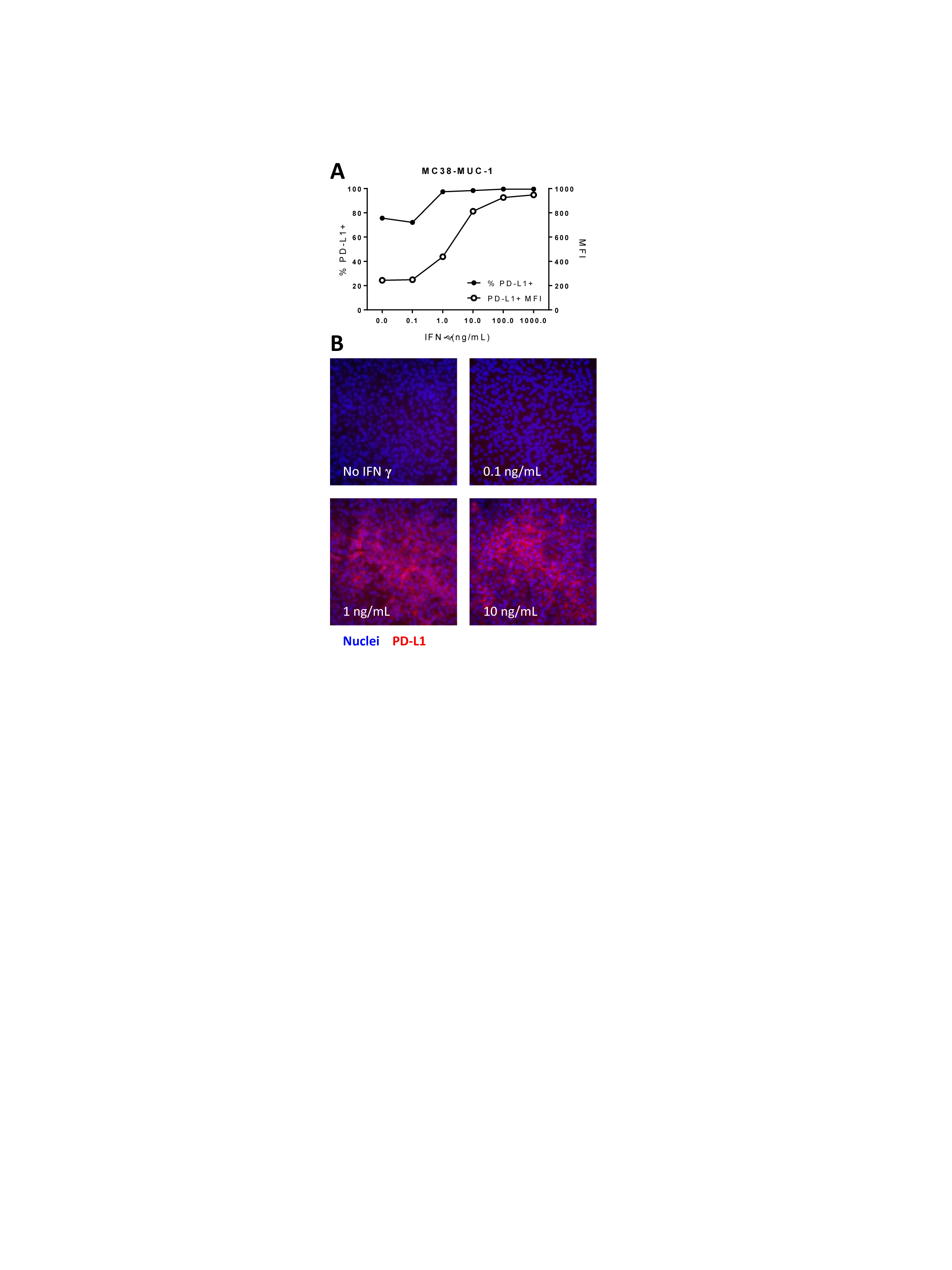

Supplement: S1 Fig — MC38-MUC1 cells were stimulated with varying concentrations of IFNγ for 18 hours. A) Percent of cells expressing PD-L1 and the mean fluorescence intensity (MFI) by flow cytometry. B) Cells were stimulated with IFNγ for 18 hours at concentrations indicated in each panel then stained for PD-L1 (red) and a nuclei stain (DAPI, blue). (TIF) [file pone.0150084.s001.tif]

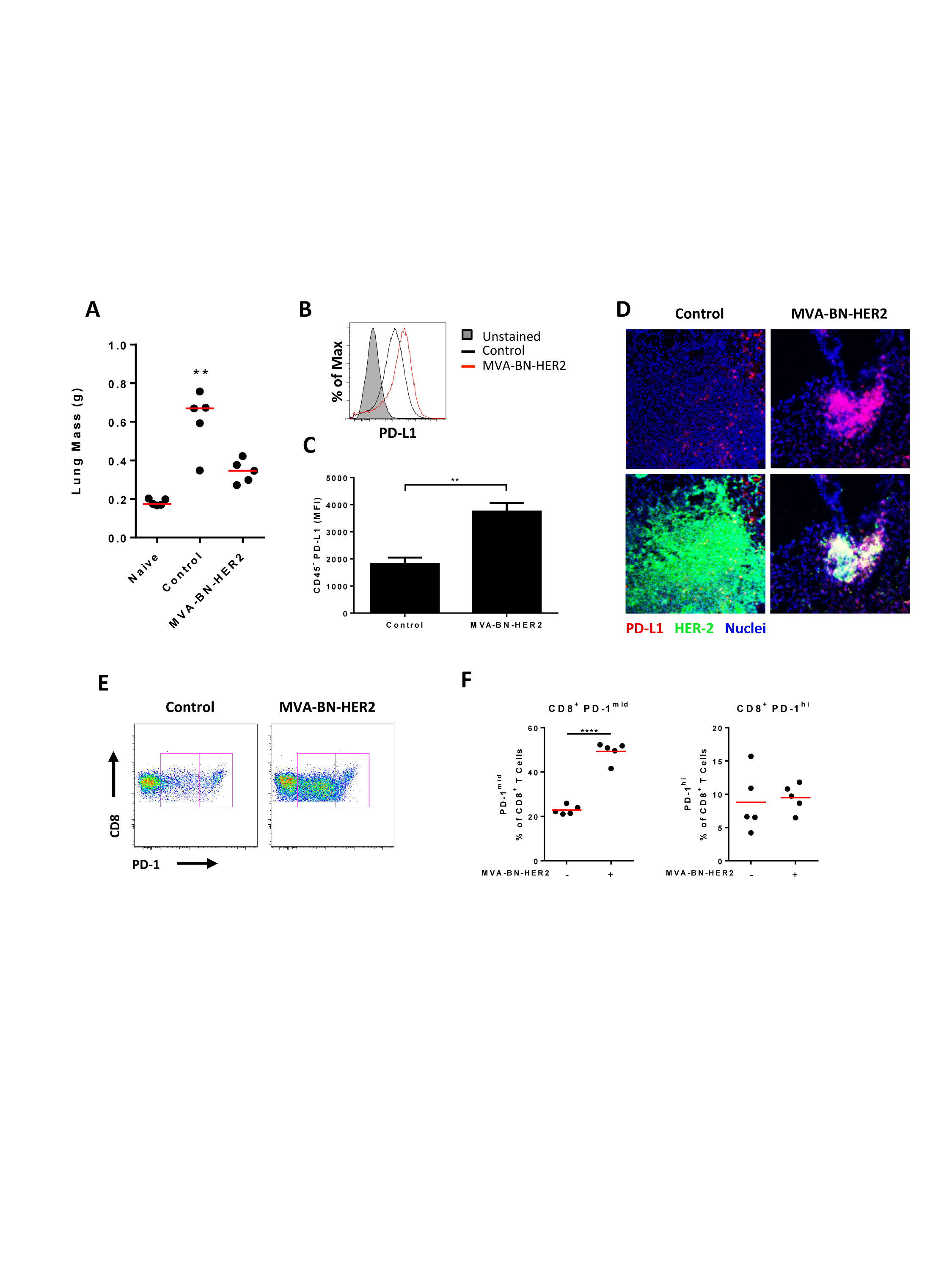

Supplement: S2 Fig — BALB/c mice were implanted with CT26-HER-2 cells (i.v.) on day 1 and treated with MVA-BN-HER2 (1E7 Inf.U) on days 4 and 11. A) Lung mass and tumor burden on day 15. B) Representative Mean Fluorescence Intensity (MFI) in control and MVA-BN-HER2 treated mice, C) Average MFI (n = 5 mice/group). ** p<0.01. D) 20 μm lung and associated tumor section with staining for HER-2 (green), PD-L1 (red) and Nuclei (blue, DAPI). E) Representative flow cytometry from control or MVA-BN-HER2 treated mice on day 15 with a CD8+ PD-1mid population (left box) and a CD8+ PD-1hi population (right box), F) Average CD8+ PD-1mid and PD-1hi expression in the lungs/tumor on day 15. (TIF) [file pone.0150084.s002.tif]

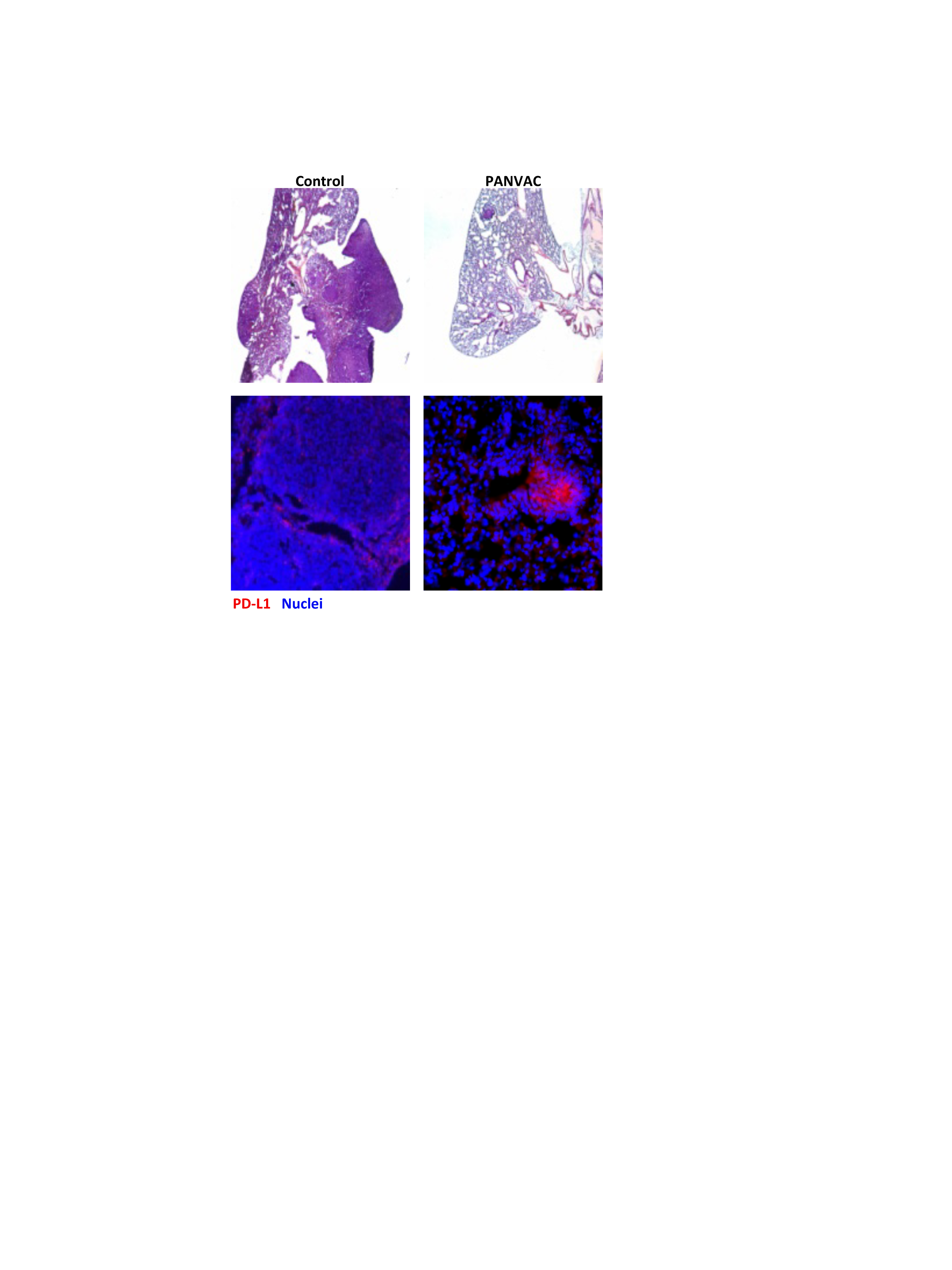

Supplement: S3 Fig — C57/BL6 mice were implanted with MC38-MUC1 cells (i.v.) on day 1 and treated with PANVAC-V (1E7 Inf.U) on day 4 and PANVAC-F (5E7 Inf.U) on days 11 and 18. On day 25 lungs/tumors were collected and stained for H&E or PD-L1. (TIF) [file pone.0150084.s003.tif]

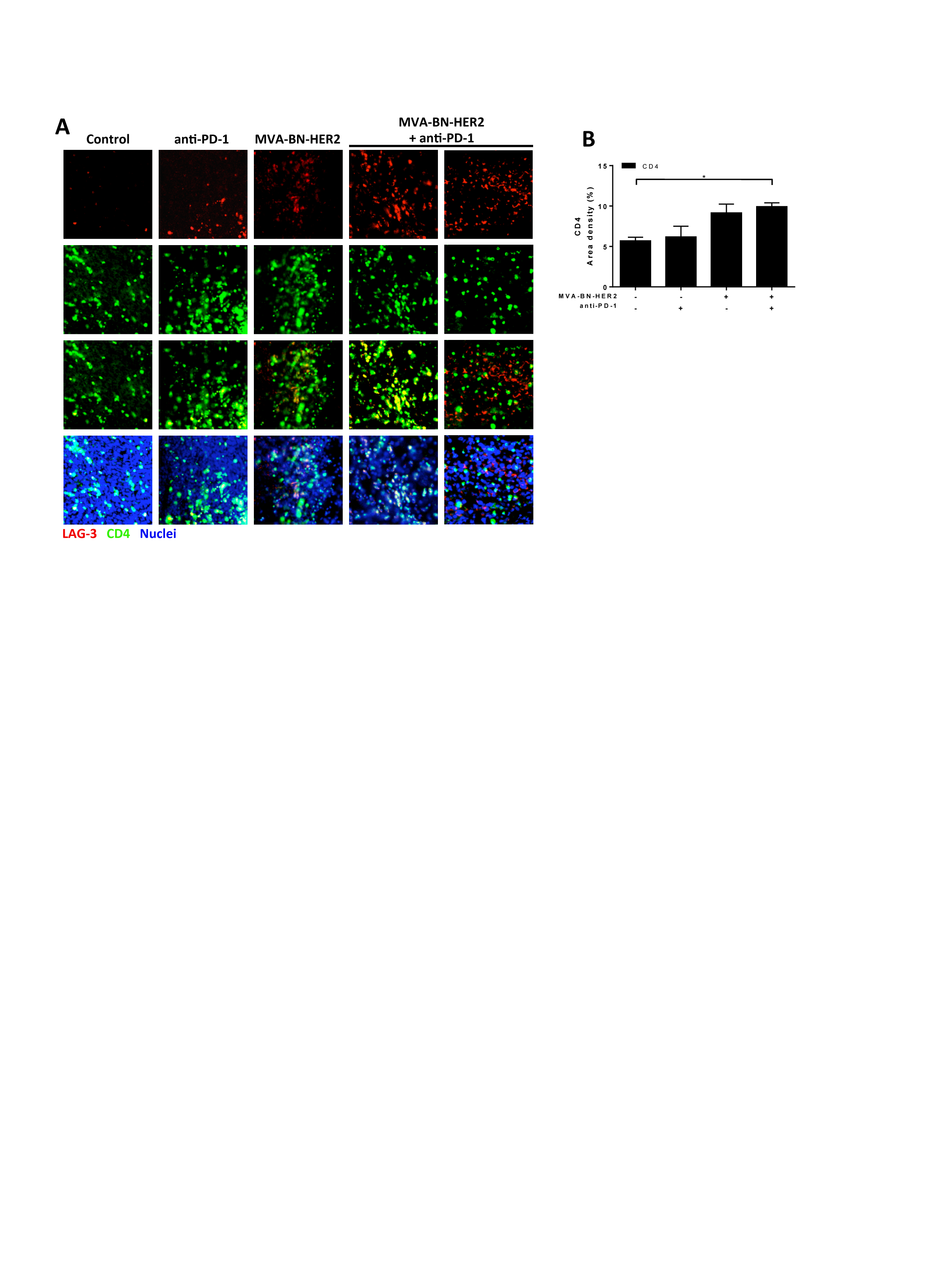

Supplement: S4 Fig — (A) Tumors from mice treated with MVA-BN-HER2 and/or anti-PD-1 were collected on day 16 and stained for LAG-3 (red), CD4 (green), and nuclei (DAPI, blue). (B) The area density for CD8+ T cells.* p<0.05. n = 3–4 mice/group. (TIF) [file pone.0150084.s004.tif]

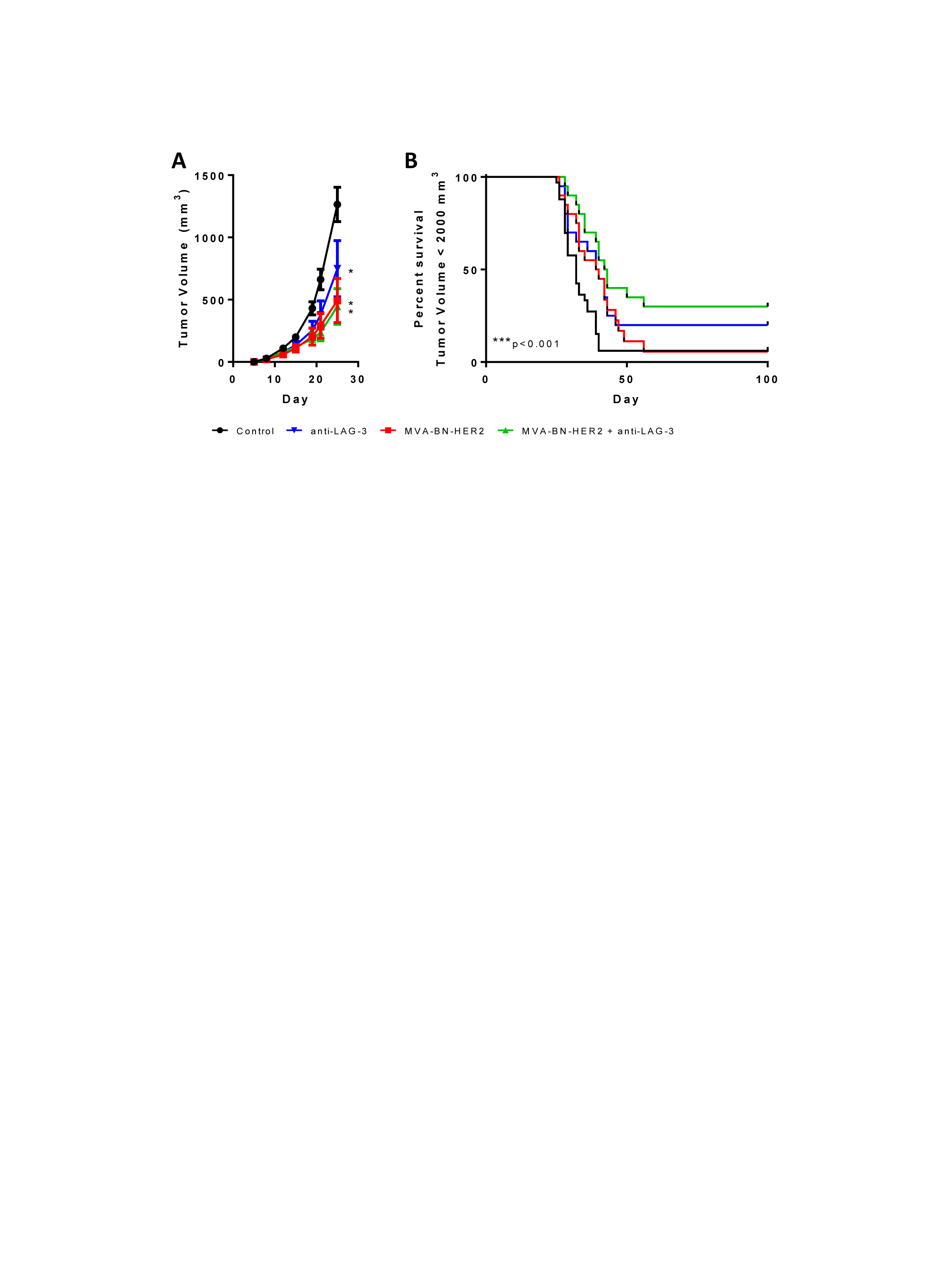

Supplement: S5 Fig — BALB/c mice were implanted with CT26-HER-2 cells on day 1 (i.d.) and treated with MVA-BN-HER2 (1E7 Inf.U) and/or anti-LAG-3 (200 μg) on days 1 and 15. A) Tumor growth with MVA-BN-HER2 and anti-LAG-3 combination therapy. C) Survival from two independent studies. Statistical significance was determined by: a two-way RM-ANOVA with Tukey’s multiple comparison test for tumor growth or a Log-rank test for survival. * p<0.0001 vs. control. (TIF) [file pone.0150084.s005.tif]
