# Supplementary material for: Poxvirus-Based Active Immunotherapy with PD-1 and LAG-3 Dual Immune Checkpoint Inhibition Overcomes Compensatory Immune Regulation, Yielding Complete Tumor Regression in Mice
Source: PLoS One. 2016 Feb 24;11(2):e0150084. doi: 10.1371/journal.pone.0150084 (PMC4765931; doi:10.1371/journal.pone.0150084)
Supplement: S1 Table — NR = not reached. BALB/c mice were implanted with CT26-HER-2 cells on day 1 (i.d.) and treated with MVA-BN-HER2 (1E7 Inf.U), anti-PD-1 (200 μg), or anti-LAG-3 (200 μg) on days 1 and 15. Survival based on tumor volume of 2000 mm3. (DOCX) [file pone.0150084.s006.docx]

**S1 Table. Median Overall Survival (mOS) and % Tumor free mice.**

| **Therapy** | **Median Survival (days)** | **% Tumor Free** |
| --- | --- | --- |
| Control | 32 | 6 (2/33) |
| MVA-BN-HER2 | 39.5 | 10 (2/20) |
| Anti-PD-1 | 35.5 | 30 (6/20) |
| Anti-LAG-3 | 39.5 | 20 (4/20) |
| Anti-PD-1 + Anti-LAG-3 | NR^a^ | 70 (14/20) |
| MVA-BN-HER2 + Anti-PD-1 | 65.5 | 45 (9/20) |
| MVA-BN-HER2 + Anti-LAG-3 | 42.5 | 30 (6/20) |
| MVA-BN-HER2 + Anti-PD-1 + Anti-LAG-3 | NR^a^ | 100 (20/20) |

BALB/c mice were implanted with CT26-HER-2 cells on day 1 (i.d.) and treated with MVA-BN-HER2 (1E7 Inf.U), anti-PD-1 (200 µg), or anti-LAG-3 (200 µg) on days 1 and 15. Survival based on tumor volume of 2000 mm^3^.

^a^NR= not reached
