# Supplementary material for: Poxvirus-Based Active Immunotherapy with PD-1 and LAG-3 Dual Immune Checkpoint Inhibition Overcomes Compensatory Immune Regulation, Yielding Complete Tumor Regression in Mice
Source: PLoS One. 2016 Feb 24;11(2):e0150084. doi: 10.1371/journal.pone.0150084 (PMC4765931; doi:10.1371/journal.pone.0150084)
Supplement: S2 Table — BALB/c mice were implanted with CT26-HER-2 cells on day 1 (i.d.) and treated with MVA-BN-HER2 and anti-PD-1 at doses indicated in the tables. Tumor growth inhibition was calculated from the untreated control. A combination index was calculated with the using the Chou-Talalay method and CompuSyn Software. (DOCX) [file pone.0150084.s007.docx]

**S2 Table. MVA-BN-HER2 synergized with PD-1 to delay tumor growth.**

| **MVA-BN-HER2 (Inf.U)** | **anti-PD-1 (µg)** | **Tumor Growth Inhibition (%)** | **Combination Index (CI)** | **Description** |
| --- | --- | --- | --- | --- |
| 0 | 0 | 0.0 |  |  |
| 1.00E+05 | 0 | 6.7 |  |  |
| 1.00E+06 | 0 | 32.8 |  |  |
| 1.00E+07 | 0 | 45.9 |  |  |
| 1.00E+07 | 0 | 71.4 |  |  |
| 0 | 22 | 28.3 |  |  |
| 0 | 66 | 14.1 |  |  |
| 0 | 200 | 61.2 |  |  |
| 1.00E+07 | 22 | 61.9 | 0.496 | Synergism |
| 1.00E+07 | 66 | 89.3 | 0.031 | Very Strong Synergism |
| 1.00E+07 | 200 | 84.1 | 0.114 | Strong Synergism |

BALB/c mice were implanted with CT26-HER-2 cells on day 1 (i.d.) and treated with MVA-BN-HER2 and anti-PD-1 at doses indicated in the tables. Tumor growth inhibition was calculated from the untreated control. A combination index was calculated with the using the Chou-Talalay method and CompuSyn Software.
